# Current Preclinical and Clinical Evidence of Shock Wave Therapy for Spinal Cord Injury: A Systematic Review

**DOI:** 10.3390/cells15080687

**Published:** 2026-04-13

**Authors:** Sofie Nagele, Berit Zellmer, Michael Graber, Leo Winter-Pölzl, Clemens Engler, Jakob Hirsch, Sophia Schmidt, Jonas Eder, Ronja Lohmann, Maria Ioannou-Nikolaidou, Vanessa Heim, Michael Grimm, Nikolaos Bonaros, Can Gollmann-Tepeköylü, Johannes Holfeld, Felix Nägele

**Affiliations:** 1 University Hospital for Cardiac Surgery, Medical University of Innsbruck, Anichstrasse 35, 6020 Innsbruck, Austria; 2 Institute for Clinical and Functional Anatomy, Medical University of Innsbruck, 6020 Innsbruck, Austria; 3 Department of Cardiovascular Surgery, German Heart Center Munich, School of Medicine & Health, Technical University of Munich, 80333 Munchen, Germany

**Keywords:** spinal cord injury, extracorporeal shock wave therapy, shock waves, nervous tissue regeneration

## Abstract

**Highlights:**

**What are the main findings?**

- Shock waves prove to be a safe and effective, non-invasive, promising treatment approach for spinal cord injury in experimental and clinical settings.

**What are the implications of the main findings?**

- Further evidence in the form of prospective trials is needed to (a) identify patient populations that benefit the most, (b) define targeted treatment protocols depending on the underlying pathology and (c) facilitate broader adoption within the community.

**Abstract:**

The diagnosis of spinal cord injury (SCI) remains associated with a poor prognosis due to limited treatment options and the absence of curative therapies. Optimizing treatment strategies is therefore crucial to enhance patients’ quality of life, reduce mortality and re-hospitalization rates, and lower overall therapy costs. Shock wave therapy (SWT) is a well-established regenerative treatment option for pathologies of the musculoskeletal system that delivers high-energy acoustic waves. SWT is non-invasive, safe and cost-effective. Preclinical and clinical evidence is emerging, showing the efficacy of SWT in the treatment of both traumatic and ischemic SCI. This systematic review synthesizes evidence on SWT in SCI from 2000 to 2025, excluding case reports and non-regenerative applications. Results were categorized into preclinical and clinical studies, with preclinical findings further divided into functional, histological, cellular, and molecular outcomes. Promising preclinical results led to initial clinical studies, which demonstrated the safety and feasibility of SWT, with a randomized controlled trial currently ongoing (ClinicalTrials.gov: NCT04474106). Overall, the encouraging evidence suggests that SWT is a promising novel regenerative treatment option for SCI, although further research is needed to define optimal treatment protocols and to establish its role in standard clinical care.

## 1. Introduction

The spinal cord, as a key component of the central nervous system, connects the brain with the peripheral nervous system, transmitting motor and sensory signals and coordinating reflexes. Each year, between 250,000 and 500,000 individuals worldwide are diagnosed with traumatic SCI (spinal cord injury). Depending on the location and severity of the lesion, SCI can result in partial or complete loss of motor and sensory function, as well as impaired autonomic regulation, including breathing, bowel, bladder, and sexual function [1].

Beyond its profound impact on patients’ quality of life, SCI imposes substantial economic burdens on individuals, families, and healthcare systems. Economic modeling analysis of direct costs for severe injuries such as high tetraplegia from the UK can escalate up to £1.12 million per SCI case. Additional ongoing costs in subsequent years, including indirect costs like lost wages, increase this value. Frequent re-hospitalizations, particularly for genitourinary, skin, respiratory, digestive, circulatory, and musculoskeletal complications, are major contributors to these expenses [2].

A variety of cells, including astrocytes, microglia, oligodendrocytes, and neurons, interact in the physiological setting of the central nervous system. After SCI, these interactions are disrupted, contributing to incomplete recovery [3].

SCI can be divided into a primary and a secondary phase, each characterized by distinct pathophysiological mechanisms. Acute SCI is caused by the actual impact on the spinal cord and marks the primary injury. Most commonly, acute SCI results from a sudden trauma to the spine and, therefore, to the spinal cord. The secondary injury phase can be subdivided into an acute, sub-acute, and chronic injury phase and is triggered by the primary reaction, resulting in further chemical and inflammatory damage to the spinal cord [4].

The acute phase of the secondary SCI is characterized by ischemia, vascular injury, hemorrhage, inflammation, and hypoxia, leading to excitotoxicity, ionic imbalance, and edema [4,5]. Sub-acute and chronic stages are dominated by neuroinflammation, oxidative stress, and apoptosis, resulting in axonal degeneration, glial scarring, and neurological dysfunction. Initially, glial scars help contain damage and facilitate limited regeneration; however, persistent scarring and upregulation of inhibitory molecules, such as chondroitin sulfate proteoglycans (CSPGs), obstruct axonal growth and cell differentiation [4,6].

Only preventive measures, like occupational safety and health efforts, can prevent the primary injury. Therefore, therapeutic interventions that influence the long-term outcome of SCI are focused on the secondary injury [7], in which a complex secondary pathophysiological cascade characterized by biochemical and physiological changes develops in the injured spinal cord and constitutes a key target for therapeutic strategies [6].

Despite extensive experimental research, effective regenerative treatments for SCI remain limited, and the translation of promising preclinical findings into clinical practice continues to be a major challenge [8,9]. This unmet need has driven the development of novel non-pharmacological, non-invasive regenerative approaches [10,11]. Among these, SWT has emerged as a promising treatment modality due to its well-established regenerative potential, safety, and cost-effectiveness [11,12].

SWT was developed from extracorporeal shock wave lithotripsy, which has been in clinical use for decades in urology and general surgery to break up kidney and gallstones [13]. The observation of an osteoblastic response, i.e., stimulation of bone-forming activity, made during animal studies in the 1980s led to an increase in interest in SWT as a non-invasive, regenerative treatment [14]. Ever since, extensive research has been conducted in the field of SWT, and numerous studies suggest a positive therapeutic effect of SWT in a wide range of pathologies. It has become clear that different tissues respond differently to SWT. Bone exhibits pronounced mechanosensitivity, where SWT-induced mechanotransduction triggers the release of growth factors. In contrast, nervous tissue shows limited mechanosensitivity; here, the therapeutic effects of SWT are primarily mediated through biochemical signaling pathways, enhanced vascularization, and modulation of inflammation driven by changes in the molecular environment. Over recent decades, SWT has proven to be safe through various indications on different organ systems. This de-risking is a major advantage of SWT compared to other experimental treatment approaches [12,13,14].

To use SWT effectively, it is important to understand the underlying basic physical principles of shock waves. A shock wave can be described as an acoustic pulse that permeates tissue and carries energy [15]. It has certain characteristics, such as high peak pressure of up to 150 MPa, a fast initial rise, a small pulse width, and a broad frequency spectrum from approximately 150 kHz up to 100 MHz [16].

Within the field of SWT, there is a distinction between radial and focused shock wave generation. Radial shock waves spread more superficially than focused shock waves and are generated via the acceleration of a projectile through compressed air until an applicator is reached. In contrast, focused shock waves are characterized by a pressure field that converges on a focal point within deeper tissue layers [17].

The generation of focused waves differs depending on the device in use. Focused shock waves can, for instance, be produced by using the electrohydraulic principle. It is the oldest way of producing shock waves in medicine. Further, shock waves can be generated by means of piezoelectric or electromagnetic principles. Piezoelectric crystals, arranged on a concave surface are electrically activated thereby rapidly deformed, generating a self-focusing pressure pulse in the surrounding fluid. Electromagnetic shock waves are emitted by rapid membrane displacement induced by a flat or cylindrical coil [18].

Depending on the intended application of the shock wave, the device is chosen appropriately. The biological effects of shock waves due to mechanical stimulation are versatile. It is reported that SWT induces a variety of processes such as angiogenesis, anti-inflammatory effects, bone remodeling, tissue regeneration, and wound healing [15].

Promising results in preclinical research paved the way for the first clinical application of shock wave therapy to treat SCIs of various etiologies. To our knowledge, there is currently no systematic review of preclinical and clinical results available. Given the growing interest in SWT as a regenerative therapy, a systematic synthesis of the available evidence is essential to clarify its therapeutic potential, identify knowledge gaps, and guide future research. The main objective of this study, therefore, is to provide a comprehensive overview of research results regarding shock wave therapy in SCIs.

## 2. Materials and Methods

This review was undertaken following the reporting standards given by the Preferred Reporting Items for Systematic Reviews and Meta-Analyses (PRISMA) 2020 statement [19]. The completed PRISMA 2020 checklist is provided in Supplementary Material Table S1. To enhance methodological transparency, the review protocol was registered on the International Platform of Registered Systematic Review and Meta-analysis Protocols (INPLASY) (registration number: INPLASY202630053).

### 2.1. Eligibility Criteria

Studies selected for this review all fulfilled the following criteria: original articles published between 2000 and 2025 in peer-reviewed journals, written in English language, and addressing the therapeutic use of SWT in SCI. Exclusion criteria comprised case reports, studies not applying shock wave therapy in a regenerative approach, studies published outside the predefined time frame, review articles, and studies with different therapeutic targets. Publications older than 2000 were excluded from the primary analysis. However, they were reviewed to provide background on the current state of research, along with review articles and studies addressing shock wave therapy in general or lacking a specific focus on spinal cord injury. When presenting the results, a primary distinction is made between preclinical and clinical studies.

### 2.2. Information Sources and Search Strategy

A systematic search was performed on PubMed and ClinicalTrials.gov to select relevant studies investigating the use of shock wave therapy in spinal cord injury. In an initial search, a total of 94 research papers were identified in PubMed using the keyword search terms (spinal cord injury) AND ((shock wave) OR (shockwave)). Additionally, eleven studies were identified through ClinicalTrials.gov using the same search terms. Scopus and Web of Science databases were cross-checked with the same search strategy, which yielded no additional results.

### 2.3. Document Selection

After the original investigation, duplicate records were removed. The remaining results were screened, retrieved, and assessed for eligibility afterwards.

Two reviewers independently assessed titles as well as abstracts for potential eligibility. Full texts were retrieved for records considered potentially relevant by both reviewers. Discrepancies during abstract screening were resolved by conducting a full-text review and involving a third reviewer. Final inclusion decisions were made by consensus in meetings with all three reviewers. Two of the included reports represented study protocols without available outcome data and were therefore not counted as separate studies in the final count.

### 2.4. Data Selection

Eligible articles were reviewed independently by two reviewers to extract the following data items for preclinical studies: type of research, study model and outcome measures categorized into functional (locomotor and sensory function), histological (morphology), cellular (cell death and angiogenesis/growth factors), and molecular outcomes (neurotrophic factors and innate immunity).

An analogous data extraction process was performed for clinical studies based on the following criteria: interventional model description, status, disease investigated, treatment, inclusion criteria and primary endpoint.

## 3. Results

**Figure 1:**
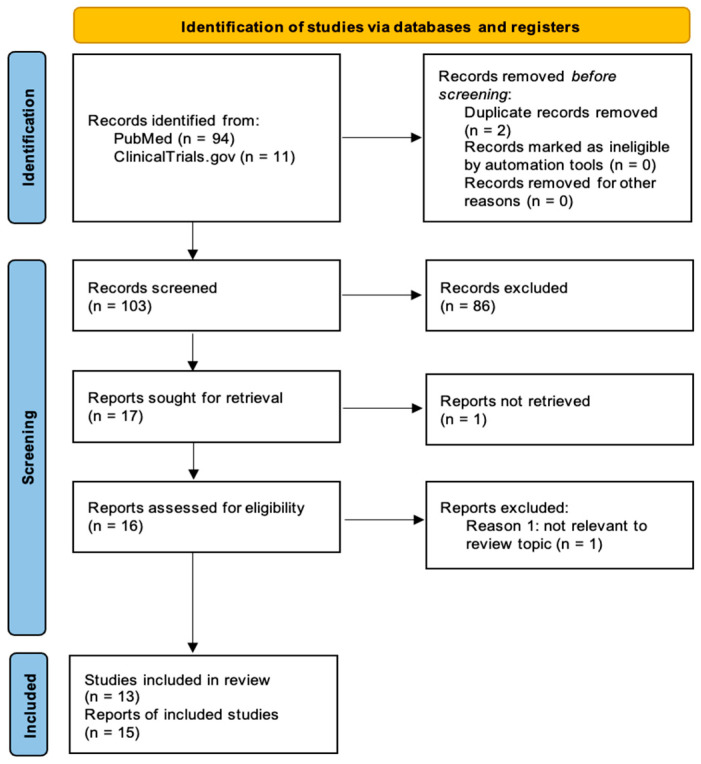
PRISMA 2020 flow diagram illustrating the stages of the literature selection.

Figure 1 illustrates the PRISMA 2020 flow diagram of document identification and screening [19].

An overview of the original articles meeting the eligibility criteria is provided in the following section. The information is structured into preclinical and clinical articles. Preclinical studies are further subdivided according to the investigated biological pathways, while clinical articles include both study protocols and completed studies.

More than half of the preclinical research projects (61.5%) were conducted with rat models. These results are in accordance with the findings of Kjell et al. [20], who discussed and elucidated that spinal cord injuries in rats have proven to be most similar to human SCIs in terms of pathology and recovery, which is essential to understand the pathophysiology and therefore to develop new therapies. For that reason, rats seem most suitable for the research on the regenerative aspect of SWT in SCI. When studying transgenic techniques and therapies in SCI, however, mice are still most used. Even though there certain progress when it comes to transgenic rats, mice are still more favorable at present [20].

While not all research results agree on the different described effects of SWT, none of the studied articles describe major damage over the course of their observational period, which ranged from 4 to 6 weeks. Specifically, hemorrhage, vacuoles, and morphological changes in neurons were considered potential adverse events and were included as outcome parameters but did not occur in these studies. Yamaya et al. also explicitly investigated the effects of shock waves applied to an uninjured spinal cord. No obvious neuronal damage could be detected. The assumption that the spinal cord remains unharmed was supported by the results of immunohistochemical staining, where no neuronal loss was detectable [21]. These results are in line with the results of Holfeld et al. and Li et al., who demonstrated in large animal models the positive effect of SWT as well as no acute or chronic adverse effects [22,23].

Over three quarters of the reviewed preclinical papers (76.9%) focused on traumatic spinal cord injury with contusion as the predominant model of trauma. The remaining studies addressed spinal cord injury of ischemic origin or investigated hypoxia and glucose deprivation in cellular experimental models. Table 1 synthesizes the findings of the preclinical studies included in the review.

### 3.1. Locomotor Function

One of the main questions, which was examined in more than three quarters of the trials, was whether SWT would affect the physical performance of rats after SCI. To classify locomotion after the intervention or non-treatment, respectively, the Basso–Beattie–Bresnahan (BBB) locomotor rating scale was used, which is well-established in preclinical trials. Matsuda et al.’s findings that locomotor function improves significantly after SWT treatment is in accordance with the results of other trials such as those conducted by Gollmann-Tepeköylü et al. and Yahata et al. [29,30,32]. Other tests to assess mobility, strength, and coordination as well as speed were performed by Matsuda et al. and Lobenwein et al. [29,33]. Their results coincide with the above-mentioned trials and strongly suggest that SWT plays a crucial role in the recovery of locomotor function after SCI. To further investigate the reason for the test results, Matsuda et al. measured the conduction of the central motor pathway by assessing the motor-evoked potentials (MEPs). While there was no significant difference in the amplitude of MEPs between the intervention and control group, the latency was significantly elongated in the control group. Poor locomotion and a long MEP latency correlated significantly, indicating that the electrophysical conductivity of the spinal cord was more preserved in the treatment group and therefore, achieved BBB scores were higher [29]. Hsu et al. examined a single SWT treatment (one week after injury) versus triple SWT treatment (one, two, and three weeks after injury) and found that triple SWT led to better motor function outcomes than a single treatment, as measured by the BBB score, print intensity, print length, average speed, and maximum variation [24].

### 3.2. Sensory Function

Improvement in not only locomotion but also sensory function has been reported by several researchers. Yahata et al. discovered that not only locomotor abilities changed but also mechanical and thermal allodynia. Animals in the SWT group had a significantly higher withdrawal threshold to mechanical stimuli than animals in the control group. The same results were seen when thermal allodynia was examined [32]. These findings coincide with Matsuda et al., who showed a significant reduction in sensory impairment induced by SWT [29]. Based on all research results, it can be presumed that the improvement in sensory function is also a consequence of more preserved electrophysical conductivity due to SWT treatment.

### 3.3. Morphology

Controversial results can be found to some extent concerning macro- and microscopic variances in SWT-induced changes to the spinal cord, which can be measured by μCT, MRI and tissue analysis. While each method has its limitations, it can be roughly said that the amount of viable tissue correlates with function.

Ashmwe et al. analyzed morphological parameters of the spinal cord after SCI in their study. They report that no significant changes could be observed between the SWT treatment and control group, using 3D parameters generated by μCT scans. Further, they conducted histological and immunohistochemical tests of the spinal cord but neither with standard H&E nor in Neurofilament and Luxol staining were morphological changes significantly different between both groups [27]. These findings completely contradict prior research results by Matsuda et al., Gollmann-Tepeköylü et al. and Yahata et al. All studies performed tissue analyses and report that decreased lesion size and spared white matter could be detected in SWT groups [29,30,32]. Furthermore, Matsuda et al. conducted an immunohistochemical analysis of neurofilament by means of RT97 antibodies and were able to detect more frequent RT97-positive fibers in the SWT group. The immunodensity of RT97-positive fibers proved to be significantly higher than that in the control group [29].

Shin demonstrated that SWT after SCI leads to proliferation of endogenous neural stem cells, especially in the ependymal region and the dorsal horn. Limited differentiation into neuronal and glial lineages was also detected. Furthermore, axonal regeneration was supported by significantly elevated GAP-43 and MAP-2 expression levels in the experimental groups compared to controls [31].

### 3.4. Cell Death

The direct effect of SWT on cell death was demonstrated in different trials. Graber et al. indicated that SWT reduced ROS production as well as apoptosis rates in SH-SY5Y cells following ischemic injury [25]. Lobenwein et al. focused on molecular processes in the context of neuronal pharmacological pre- and postconditioning and SWT as treatments for spinal cord ischemia. They used apoptosis markers such as Caspase3 and poly (ADP-ribose) polymerase1 (PARP-1) and their active, apoptosis-inducing forms. Apoptosis indicators could be reduced pharmacologically via a TLR-3 agonist, and decreased levels could be achieved via SWT in a postinjury setting. Neuronal apoptosis was therefore directly influenced and reduced by shock wave application [26]. Lobenwein et al. already demonstrated in an earlier study that SWT affects neuronal degeneration directly. As a model for spinal cord ischemia, mice were used as well as spinal slice cultures ex vivo. While neuronal degeneration proceeded up to 7 days after spinal cord ischemia in the control group, the number of degenerating neurons remained stable after 24 h in the intervention group [33]. These results fit with Yahata et al.’s findings that the quantity of TUNEL (terminal deoxynucleotidyl transferase dUTP nick end labeling)-positive cells, another apoptosis indicator, was significantly lower at the sides (1000 μm rostral and caudal) as well as at the center of the injury itself after SWT completed 7 days after injury [32]. Yamaya et al. analyzed the loss of neuronal tissue by neuronal nuclei (NeuN) staining samples 42 days after injury and found out that the shock wave treatment group had a significantly higher number of NeuN-positive cells than the control group. Significant changes were observed in the center as well as 1000 μm rostral [21]. These findings are in line with Gollmann-Tepeköylü et al., who demonstrated a significantly reduced number of degenerating neurons in vitro as well as in murine and human tissue induced by SWT [30].

Aside from that, it has been demonstrated in several studies that underlying molecular mechanisms play a crucial role in preventing apoptosis and inducing regeneration.

### 3.5. Molecular Mechanisms

Because shock wave treatment was originally established for skeletal disorders, there exists a substantial number of molecular studies in this field compared to non-skeletal applications. It has been reported in multiple studies that shock waves have a positive effect on neovascularization, anti-inflammation, regeneration of tissue and osteogenesis, and chondroprotection. The molecular changes attributable, at least in part, to SWT are complex and involve the up- and downregulation of numerous signaling factors. A detailed overview of the up- and downregulation of various factors is illustrated in a review about SWT in musculoskeletal disorders by Moya et al. [36]. With the evidence of several previously mentioned studies, it can be concluded that SWT also has an impact on molecular factors that are crucial for spinal cord regeneration.

However, the interpretation of molecular findings across studies is limited by considerable variability in experimental sampling time points. As molecular responses to SWT appear to occur within relatively short time frames, differences in sampling strategies may result in substantially divergent or even partially contradictory findings. For instance, Lobenwein et al. collected samples shortly after treatment (30 min to 24 h) following oxygen deprivation in cell cultures, whereas Matsuda et al. evaluated molecular changes 7 and 21 days after injury with repeated treatments over three weeks. In contrast, Pastor et al. analyzed samples 35 days after injury, corresponding to one week after the final treatment [26,28,29]. Despite these methodological differences, the available evidence consistently indicates that SWT modulates molecular pathways relevant to spinal cord regeneration.

#### 3.5.1. Angiogenesis and Growth Factors: VEGF, HIF-1α, FGF1, and FGF2

Prior research on SWT in the context of skeletal disorder treatment has shown that the microenvironment of cells changes in response to shock wave application. One of the most prominent differences involves the vascular endothelial growth factor (*Vegf*). Yamaya et al. demonstrated impressively that mRNA expression of *Vegf* and its receptor fms-like tyrosine kinase 1 (*Flt1*) was significantly higher in the treatment group than in the control group. Further, they verified their results by investigating the immunodensity of VEGF, which was significantly different as well between the control and intervention group. Both results were documented 7 days after treatment [21]. That coincides with Lobenwein et al.’s results. They measured mRNA expression of *Vegf* and hypoxia-inducible factor 1 alpha (*Hif1a*) 24 h and 48 h, respectively, after ischemia in a treatment and control group. Expression of mRNA of both factors was significantly higher after SWT both times. To confirm angiogenesis, they performed immunostaining for CD31-positive capillaries and discovered a significant increase in capillaries in the treatment group [33]. Similar results were reported by Yahata et al. and Pastor et al. [28,32]. Yahata et al. demonstrated *Vegf* expression in different cells, such as neurons, astrocytes, and oligodendrocytes [32]. Pastor et al., however, only reported an immediate increase in *Vegf* after recent SCI and a surge in *Vegf* in mice with chronic SCI [28].

Hsu et al. focused on another class of growth factors, specifically fibroblast growth factors 1 and 2 (*Fgf1* and *Fgf2*). Shock wave treatment reduced the elevated expression of inflammation-induced *Fgf1*, *Fgf2*, *Fgfr1*, and *ERK* in SCI rats. Triple SWT was more effective than single SWT, suggesting that shock wave therapy protects the injured spinal cord by modulating *Fgf* and *Erk* signaling involved in inflammation and tissue repair [24].

#### 3.5.2. Neurotrophic Factors: *Nt3* and *Bdnf*

*Nt3* is a neurotrophin and is released by the nervous system after injury to enable regeneration and survival. Lee et al. investigated functional recovery and neurotrophin-3 expression (*Nt3*) in the spinal cord after peripheral nerve injury (sciatic nerve). It was demonstrated that *Nt3* levels were significantly increased one day after injury in the SWT treatment group as compared to a control group. Furthermore, it was shown that early treatment increased *Nt3* and *Nt3* mRNA and had an impact on macrophages and Schwann cells, which in turn influence the possibility of neurons to regenerate [34].

Brain-derived neurotrophic factor (*Bdnf*) also belongs to a group of neurotrophins that regulate function, differentiation, and survival of neurons as mentioned by Matsuda et al. and Huang et al. [29,37]. Lee et al. demonstrated that *Bdnf* expression was significantly higher in the SWT groups than in the control group 6 weeks after SCI [35]. Matsuda et al. focused especially on the expression of *Bdnf*. They reported a significantly higher expression of *Bdnf* mRNA 7 days after SCI in the intervention group in comparison to the control group. To further investigate the results, they compared the expression of Tropomyosin receptor kinase B (*Ntrk2*), a receptor for neurotrophins, in both groups. The SWT group showed a significant increase 7 days after SCI. At day 21, however, there were no more statistically significant differences between the groups concerning *Bdnf* mRNA and *Ntrk2*. To verify the results, Matsuda et al. measured the actual *Bdnf* expression and confirmed the significantly increased upregulation of *Bdnf* as well as immunodensity. Additionally, it was possible to prove that *Bdnf* was expressed in different cell cultures such as neurons, astrocytes, and oligodendrocytes [29].

#### 3.5.3. Innate Immunity

Inflammation, which occurs after SCI, is a complex process with numerous involved factors as described by Alizadeh et al. [6]. More than a decade ago, Davis et al. demonstrated that shock waves modulate inflammation in epidermal tissue in mice, which triggered several following studies exploring immunological mechanisms induced by shock waves [38]. Studies conducted by Lobenwein et al., Gollmann-Tepeköylü et al. and Graber et al. examined the influence of shock waves on immunological factors in SCI [25,26,30,33]. Based on current knowledge at that time, Lobenwein et al. measured interleukin-6 (*Il6*) mRNA. *Il6* is known to be a pro-inflammatory cytokine and macrophage attractor. *Il6* levels were significantly increased in the intervention group after 24 h, which characterizes early inflammation. Furthermore, the amount of transforming growth factor β (*Tgfb1*), a factor that activates macrophages, was also significantly higher after shock wave application 24 h after treatment. Both surges dissolved after 48 h. The control group, however, presented the first high increase in *Il6* initially after 48 h [33]. These findings coincide with Gollmann-Tepeköylü et al. They reported a significantly higher increase in *Il6* mRNA two hours after treatment in the SWT group. Furthermore, Gollmann-Tepeköylü et al. assumed that the *Il6* surge might recruit NPCs. The supposition was verified by a quantification of cells that expressed the neuroectodermal stem cell marker nestin. It was shown that the increase in nestin-positive cells was significantly higher in the treatment group than in the control group. Gollmann-Tepeköylü et al. also detected a significant increase in nestin-positive cells after SWT. Hence, progenitor cells from the spinal cord of wild-type and *Tlr3*^-/-^ mice were isolated to do more tests. The progenitor cells taken were positive for nestin and *Tlr3*. After exposure to SWT and a *Tlr3* agonist, no proliferation could be observed in any group. However, it was possible to detect significantly increased differentiation upon exposure to SWT and a *Tlr3* agonist, which shows that differentiation seems to be *Tlr3* dependent [30].

These findings are in accordance with the newest research results by Lobenwein et al. It was observed that activated *Tlr3* could significantly reduce neuronal apoptosis. Furthermore, it was shown that both SWT or a pharmacological *Tlr3* activation could change the genetic expression of the immune response. While the *Tlr3* agonist caused a steady and significant increase in cytokines, SWT produced short and singular peaks, which might be due to different mechanisms of action [26].

*Tlr3* upregulates *Il6* via *Trif* (TIR domain-containing adapter-inducing IFN-β) and *Nfkb1* (nuclear factor k-light-chain-enhancer of activated B cells) [39]. The fact that *Tlr3* plays an important role in the process of immune response raised the question of whether neuronal survival and regeneration is *Tlr3* -dependent. This question was also addressed by Lobenwein et al. and Gollmann-Tepeköylü et al. In prior research, it was demonstrated that the effect of shock waves is *Tlr3* -dependent [40]. Lobenwein et al. were able to demonstrate that significantly fewer neurons degenerated in wild-type mice that were treated with shock waves than in *Tlr3*^-/-^ mice. Furthermore, it was shown that *Tlr4*, which shares a common signal pathway with *Tlr3*, was not unaffected by SWT but downregulated [33,39,40]. Gollmann-Tepeköylü et al. continued to investigate the role of *Tlr3*. In a model of SCI in zebrafish, they observed that locomotion was improved after *Tlr3* stimulation, whereas it was impaired when TLR3 was inhibited. These results suggest that *Tlr3* is neuroprotective [30]. The assumption was confirmed by Graber et al. by creating a transgenic *Tlr3* knockout zebrafish. After SCI, the wild-type as well as *Tlr3*^-/-^ zebrafish were treated with shock waves. It was demonstrated that the *Tlr3*^-/-^ fish showed a decreased volume of neurons within the field of injury [25]. These results display once more the outstanding importance of *Tlr3* in the context of SWT in SCI.

### 3.6. Clinical Data on the Effect of Shock Wave Therapy in Spinal Cord Injury

Based on promising results from preclinical in vitro and in vivo studies demonstrating that SWT affects the expression of pro- and anti-inflammatory cytokines, its translation into clinical applications was initiated [41,42]. The first in-human trials have been conducted or are ongoing.

#### 3.6.1. Safety and Feasibility

After demonstrating the positive effects of SWT in prior research projects, a first attempt was made to transfer preclinical research into a clinical setting. A trial was initiated in 2015, in which SWT was applied to patients with chronic complete paraplegia at the thoracic level. After the inclusion of 34 patients, no severe adverse events were observed, as reported later by Leister et al. [43].

Graber et al. conducted the first in-human trial to prove the safety as well as feasibility of SWT in patients with ischemic SCI. The trial included five patients that were treated with SWT after suffering from spinal cord ischemia. Treatment was performed using an extracorporeal shock wave device (Flashwave, Konstanz, Germany), delivering 1000 impulses (0.38 mJ/mm^2^, 5 Hz) to both paravertebral sites. The therapy covered five segments above and below the identified level of injury and was administered once weekly for six weeks. Before treatment, a baseline analysis was performed, including the American Spinal Injury Association (ASIA) impairment scale (AIS), Spinal Cord Independence Measure (SCIM), and World Health Organization Quality of Life (WHOQOL questionnaire). The treatment consisted of SWT once a week for 6 weeks. Endpoint follow-up was conducted 6 months after the first therapy and included an MRI scan, the ASIA scale, the SCIM score, and the WHOQOL questionnaire. All mentioned parameters showed satisfying results after follow-up. Importantly, no adverse events of SWT could be detected [25]. These results coincide with interim results of the mentioned trial [43] and prior results of numerous studies in the field of shock wave administration. Therefore, the application of shock waves can be considered a safe treatment based on current knowledge.

#### 3.6.2. Effects on Patients’ Quality of Life

In addition to safety and feasibility, Graber et al. investigated the impact of SWT on patients’ quality of life, assessed using SCIM and WHOQOL. The compiled SCIM score at the 6-month follow-up is significantly higher than the baseline score. The score includes aspects such as self-care, mobility indoors and outdoors as well as in rooms and the toilet, respiration, and sphincter management. Furthermore, the results of the WHOQOL show an increase in scores regarding environment and physical health [25]. Considering that the impairment of daily routines after SCI is a high burden for affected patients, an improvement in the quality of life seems clinically meaningful.

#### 3.6.3. Clinical Prospects

Since beneficial effects have been demonstrated by numerous studies, SWT seems to represent a promising treatment option for a broad variety of diseases. Due to the growing amount of positive research results, clinical implementation in a wider field seems to be a matter of time. The first major prospective, multi-center, randomized, placebo-controlled clinical trial regarding the effect of SWT in spinal cord injury was initiated by Leister et al. in July 2020. The primary endpoint is an improvement in motor and sensor functions after SWT, which will be measured with sub-scores 6 months after treatment [43]. Given that Graber et al. demonstrated the safety and feasibility of SWT, as well as measurable effects on patients’ quality of life, the results of the study by Leister et al. are anticipated with interest [25].

Table 2 summarizes the key characteristics of the included clinical study protocols and clinical studies.

## 4. Discussion

While all research projects analyze the regeneration of the spinal cord and some remarkable knowledge could be gained, it should be noted that the experiments and their conduction differ considerably. Since there is no standardized approach, each research project has its own setup, time frame, including the interval between injury and treatment, and sampling dates. Given that SWT effects on a molecular level seem to have a relatively short time frame for sampling, which has been demonstrated in several studies, results can differ significantly from each other and lead partly to contradicting conclusions. For reasons of comparability and to ascertain or dismiss hypotheses, a more standardized approach would be useful. As shown above, most studies were conducted with rats; however, the model as well as the way SCI is induced might also have an influence on the outcome and the conclusions that are drawn from the results.

It has been shown in several research projects that SWT has a positive effect on locomotion. While it is suggested that *Vegf* plays a crucial role in the improvement in locomotion and the preservation of fibers, it remains unclear whether there are other, still unknown molecular changes that can also play a major role in this improvement [21,29,30,32,33]. Ashmwe et al. focused on circulating miRNA after SWT and were able to observe an increase in circulating miR-375 and a decreased amount of miR-382-p (samples drawn 1 h before and after treatment), which might potentially be involved in the observed changes in locomotion and need to be further investigated [27].

It has also been demonstrated that SWT has a neuroprotective function. Molecular factors such as *Bdnf* and *Tlr3* are directly influenced via shock wave application. The neuroprotective function of *Bdnf* has been investigated in multiple trials, whereas the role of *Tlr3* as a neuroprotective enhancer seems relatively new [29,30,35]. Since it has already been reported that a synergistic effect of *Bdnf* and other signal proteins such as fibroblast growth factor 2 (*Fgf2*) was observed in functional dopaminergic neurons, the question arises of whether any synergistic effects of *Bdnf* and *Tlr3* exist [45]. Since the mechanisms of SWT are not yet fully understood, more important molecular factors could be involved. Moreover, the role of *Tlr4* as part of the inflammatory process is also a topic of current research and could be another key part. Pharmacological attempts to interfere and modulate these immunological cascades are part of ongoing research projects and could potentially offer new perspectives [46].

In a direct approach, Graber et al. attempted to translate preclinical findings into a clinical setting and reported promising results. They were able to demonstrate the safety and feasibility of SWT, which is a key factor to encourage and expand further investigations. Nevertheless, the findings of that study should be interpreted with caution given the small sample size and the heterogeneous composition of the study cohort. The nature of the injury was ischemic and not traumatic, which is not highly relevant when it comes to safety and feasibility but might lead to different results in terms of functional outcome and can therefore not be directly transferred [25]. Larger trials will be required to establish the efficacy of SWT. At the current stage, studies with small cohorts primarily provide evidence regarding safety and feasibility. In this context, the inclusion of a heterogeneous patient population may reflect the broader clinical spectrum of the indication.

Still, the current clinical evidence remains sparse. Besides that completed study, only two other registered clinical trials could be identified, of which one is still recruiting participants [43] and the other is listed with an unknown status and has not reported any results to date [44]. Consequently, no additional outcome data are currently available to support or challenge the preliminary findings. This limited availability of completed clinical trials substantially restricts the strength and generalizability of the conclusions that can be drawn at this stage.

One notable area of inconsistency among the preclinical studies concerns morphological outcomes following shock wave therapy. While several studies reported beneficial structural changes, such as reduced lesion size and preservation of white matter, other investigations did not observe significant morphological differences between treatment and control groups. These discrepancies may be explained by methodological differences across studies, including variations in animal models, injury induction techniques, shock wave treatment protocols, and the timing and methods of morphological assessment. Additionally, differences in imaging and histological analysis techniques may influence the detection of subtle structural changes. These factors highlight the importance of standardized experimental designs and assessment protocols in future studies to improve comparability and to better clarify the structural effects of shock wave therapy in spinal cord injury.

Future well-designed studies with larger and more homogeneous patient populations are therefore needed to validate these early findings and to determine the true therapeutic potential of SWT in SCI in a clinical setting.

In summary, the present overview of research results concerning effects of SWT in spinal cord injury shows the ongoing interest and various attempts to extend the use of shock waves in a regenerative way in spinal cord injuries. As described, SWT has been used for decades in numerous medical fields as a reasonable therapy option. In some groundbreaking trials, it was demonstrated that SWT can be regarded as a safe and feasible treatment of spinal cord injuries. Given that the first trials show no safety concerns as well as encouraging improvements, there seem to be no major obstacles to increasing the number of patients participating in trials as well as extending the study sites.

Although several molecular mechanisms potentially underlying the effects of ESWT have been proposed, including pathways involving *Tlr3* activation as well as increased expression of neurotrophic and angiogenic factors such as *Bdnf* and *Vegf*, the translational relevance of these findings remains uncertain. Most available evidence is derived from preclinical studies, and the extent to which these mechanisms contribute to therapeutic effects in humans has not yet been clearly established.

This study has limitations. Specifically, a quantitative meta-analysis was not performed due to the substantial methodological heterogeneity among the included studies. The studies differed considerably in their experimental designs, including variations in experimental models (in vivo and in vitro), species used, injury paradigms, and ESWT treatment protocols such as energy flux density, number of shocks, and timing of application. In addition, outcome measures were highly variable, ranging from functional assessments to histological, inflammatory, and angiogenic markers, often evaluated using different methodologies and time points. This variability limited the direct comparability of results across studies. Consequently, pooling the available data in a meta-analysis could have produced misleading conclusions; therefore, the evidence was synthesized using a qualitative approach. Future research should therefore aim to establish standardized experimental protocols and reporting frameworks to improve comparability between studies. Additionally, further investigation of the underlying molecular mechanisms of shock wave therapy may help to better understand its neuroprotective and regenerative effects. Importantly, large, well-designed prospective clinical trials are required to validate the safety and efficacy of shock wave therapy and to identify the patient populations and treatment parameters that may benefit most from this therapeutic approach.

## 5. Conclusions

SWT is a promising non-invasive tool serving an unmet clinical need in the treatment of spinal cord injuries of various etiologies. Preclinical evidence is growing and provides valuable insights into the underlying mechanisms. Considering that the underlying molecular mechanisms have not yet been fully understood, a causal relationship between the specific parameters and the observed outcomes can only be hypothesized at present and should be further investigated in future studies. Nevertheless, current evidence suggests that the molecular effects of shock wave therapy may be detectable within a relatively short time window following application. Future research should focus on the translation of these findings into clinical application, especially in the form of large prospective trials, to evaluate the efficacy and identify both patient groups and time points that are most likely to benefit from SWT.

## Figures and Tables

**Table 1:** Preclinical. Summary of experimental models, biological pathways and disease contexts in studies investigating the effects of SWT preclinically. Research spans basic and translational approaches using in vivo, in vitro, and ex vivo systems to assess functional, morphological, immune, and regenerative responses following traumatic, ischemic, and hypoxic injury.

Reference	Type of Research	Model	Pathway Investigated	Disease Investigated	Treatment Parameters
Hsu et al. [24]	basic	rats	locomotor function, morphology, cell death, angiogenesis and growth factor, innate immunity	traumatic—contusion	1, 2 and 3 weeks post SCI 0.13 mJ/mm^2^, 4 Hz, 500 impulses
Graber et al. [25]	translational	in vitro, zebrafish	cell death, innate immunity	hypoxic—no glucose, traumatic—contusion	1, 2, 3, 4, 5 and 6 weeks post SCI 0.38 mJ/mm^2^, 5 Hz, 1000 impulses
Lobenwein et al. [26]	basic	in vitro, mice	cell death, innate immunity	hypoxic—low glucose, ischemic	1 h prior to OGD and immediately after OGD 0.08 mJ/mm^2^, 3 Hz, 200 impulses
Ashmwe et al. [27]	basic	rats	locomotor function, morphology	traumatic—contusion	2, 3 and 4 weeks post SCI or 5, 6 and 7 weeks post SCI 1.1 J/mm^2^, 5 Hz, 500 impulses
Pastor et al. [28]	basic	mice	angiogenesis and growth factor	traumatic—contusion	3 sessions per week, 1, 2 and 3 weeks post SCI 0.11 mJ/mm^2^, 4 Hz, 500 impulses
Matsuda et al. [29]	basic	rats	locomotor and sensory function, morphology, neurotrophic factors	traumatic—contusion	0, 2, 4, 7, 9, 11, 14, 16, and 18 days post SCI 0.25 mJ/mm^2^, 4 Hz, 400 impulses
Gollmann-Tepeköylü et al. [30]	basic	mice, zebrafish, human cells ex vivo	locomotor function, morphology, cell death, innate immunity	traumatic—contusion	In vitro: single application 0.08 mJ/mm^2^, 3 Hz, 300 impulses In vivo: 2 weeks post SCI 0.1 mJ/mm^2^, 5 Hz, 500 impulses
Shin et al. [31]	basic	rats	locomotor function, morphology	traumatic—contusion	4 weeks post SCI 0.04 mJ/mm^2^, up to 2500 impulses
Yahata et al. [32]	basic	rats	locomotor and sensory function, morphology, cell death, angiogenesis and growth factor	traumatic—contusion	0, 2, 4, 7, 9, 11, 14, 16, and 18 days post SCI 0.25 mJ/mm^2^, 4 Hz, 400 impulses
Lobenwein et al. [33]	basic	mice, human cells ex vivo	locomotor function, cell death, angiogenesis and growth factor, innate immunity	ischemic	In vivo: immediately post SCI 0.1 mJ/mm^2^, 5 Hz, 500 impulses In vitro: single application 0.08 mJ/mm^2^, 3 Hz, 300 impulses
Lee et al. [34]	basic	rats	locomotor function, neurotrophic factor	traumatic peripheral nerve injury—contusion	Immediately post SCI, 10 times over the course of two weeks 0.09 mJ/mm^2^, 3 Hz, 300 impulses
Lee et al. [35]	basic	rats	locomotor function, neurotrophic factor	traumatic—contusion	4 weeks post SCI 0.04 mJ/mm^2^, 1000 impulses
Yamaya et al. [21]	basic	rats	locomotor function, cell death, angiogenesis and growth factor	traumatic—contusion	0, 2, 4, 7, 9, 11, 14, 16, and 18 days post SCI 0.1 mJ/mm^2^ 4 Hz, 400 impulses

**Table 2:** Clinical. Overview of intervention models, recruitment status and disease context in study protocols and studies addressing SWT clinically including treatment parameters and eligibility criteria as well as primary endpoints.

Reference	Type of Research	Interventional Model Description	Status	Disease Investigated	Treatment	Inclusion Criteria	Primary Endpoint
Graber et al. [25]	translational	single-arm, prospective, single-center, open-label interventional study	completed	spinal cord ischemia	level of the lesion including five cranial and five distal segments applied once a week for six consecutive weeks	suffering from spinal cord ischemia after aortic surgery clinical diagnosis of spinal cord ischemia confirmed via neurological examination and spinal MRI	assessment of ASIA impairment scale, evaluation of SCIM score and WHOQOL questionnaire after 6 months
Leister et al. NCT04474106 [43]	clinical—study protocol	two-arm three-stage adaptive, prospective, multi-center, randomized, double-blinded, placebo-controlled clinical trial	recruiting	acute traumatic SCI	level of the lesion including five cranial and five distal segments, as well as on the plantar surface of both feet, applied peri-operatively under general anesthesia (ideally immediately post-operatively after skin closure) or within the first 48 h after trauma	patients with acute traumatic spinal injuries who are awake, responsive, and oriented at admission patients from the age of 18 years admission to hospital within 24 h after injury signed informed consent participation in the Austrian Spinal Cord Injury Study (ASCIS)-Registry (only for the Austrian hospitals)	the degree of motor and sensory impairment in the AIS and the International Standards for Neurological Classification of Spinal Cord Injury (ISNCSCI) Score after 6 months
NCT03399968 [44]	clinical—study protocol	prospective, randomized, controlled, double-blinded, clinical intervention study	unknown	chronic paraplegia	level of the lesion including five cranial and five distal segments paravertebrally left and right applied once a week over 6 weeks	patients with a spinal cord injury classified as ASI A between the level of THII to THX (complete central lesion) at least 1 year after trauma no signs of spontaneous recovery over the last 6 months signed informed consent	motor function of the lower limbs assessed by gait analysis tool (Lokomat (R)) after 24 weeks

## Data Availability

The original contributions presented in this study are included in this article. Further inquiries can be directed to the corresponding author.

## Supplementary Materials

The following supporting information can be downloaded at: https://www.mdpi.com/article/10.3390/cells15080687/s1, Table S1: PRISMA checklist 2020.

## Abbreviations

The following abbreviations are used in this manuscript:

AIS	ASIA Impairment Scale
ASIA	American Spinal Injury Association
ASCIS	Austrian Spinal Cord Injury Study
BBB	Basso–Beattie–Bresnahan (Locomotor Rating Scale)
BDNF	Brain-Derived Neurotrophic Factor
CSPGs	Chondroitin Sulfate Proteoglycans
FGF1	Fibroblast Growth Factor 1
FGF2	Fibroblast Growth Factor 2
FGFR1	Fibroblast Growth Factor Receptor 1
GAP-43	Growth-Associated Protein 43
H&E	Hematoxylin and Eosin
HIF-1α	Hypoxia-Inducible Factor 1 Alpha
IL-6	Interleukin-6
ISNCSCI	International Standards for Neurological Classification of Spinal Cord Injury
MEPs	Motor-Evoked Potentials
MRI	Magnetic Resonance Imaging
μCT	Micro-Computed Tomography
MAP-2	Microtubule-Associated Protein 2
MPa	Megapascal
mRNA	Messenger Ribonucleic Acid
NeuN	Neuronal Nuclei
NF-κB	Nuclear Factor Kappa-Light-Chain-Enhancer of Activated B Cells
NT-3	Neurotrophin-3
PARP-1	Poly (ADP-Ribose) Polymerase 1
PRISMA	Preferred Reporting Items for Systematic Reviews and Meta-Analyses
ROS	Reactive Oxygen Species
RT97	Neurofilament Antibody Marker
SCI	Spinal Cord Injury
SCIM	Spinal Cord Independence Measure
SH-SY5Y	Human Neuroblastoma Cell Line
SWT	Shock Wave Therapy
TGF-β	Transforming Growth Factor Beta
TLR3	Toll-Like Receptor 3
TLR4	Toll-Like Receptor 4
TRIF	TIR-Domain-Containing Adapter-Inducing Interferon-β
VEGF	Vascular Endothelial Growth Factor
WHOQOL	World Health Organization Quality of Life
